# Regeneration of the Periodontal Apparatus in Aggressive Periodontitis Patients

**DOI:** 10.3390/dj7010029

**Published:** 2019-03-08

**Authors:** Zvi Artzi, Shiran Sudri, Ori Platner, Avital Kozlovsky

**Affiliations:** Department of Periodontology and Oral Implantology, Tel Aviv University, Tel Aviv-Yafo 69979, Israel; sudri.shiran@gmail.com (S.S.); ori.platner@gmail.com (O.P.); kavital@tauex.tau.ac.il (A.K.)

**Keywords:** periodontal regeneration, aggressive periodontitis, deproteinized bovine bone, enamel matrix derivatives (Emdogain^®^), guided tissue regeneration (GTR)

## Abstract

The purpose of this study is to evaluate and compare, retrospectively, the outcome of two different periodontal regeneration procedures in patients suffering from aggressive periodontitis (AgP). Twenty-eight patients were diagnosed with AgP, suffering from several intra-bony defects (IBD); that were treated by one of two periodontal regeneration techniques randomly assigned to each patient: a. guided tissue regeneration (GTR) or b. an application of extracted enamel matrix derivatives (EMD) combined with demineralized bone xenograft particles (DBX). Probing pocket depth (PPD), clinical attachment level (CAL), and gingival recession were recorded. Pre-treatment and follow-up (up to 10 years from the surgery) recordings were analyzed statistically within and between groups. A significant reduction was shown at time on PPD and CAL values, however, not between subject groups. CAL values decreased in all sites. At the EMD group (44 sites), CAL gain was 1.92 mm (±1.68) from pre-treatment to follow-up (p < 0.001) and at the GTR group (12 sites) CAL gain of 2.27 (±1.82) mm. In conclusion, 1–10 years observations have shown that surgical treatment of AgP patients by either GTR or by application of EMD/DBX results in similar successful clinical results.

## 1. Introduction

Aggressive periodontitis (AgP) is a periodontal disease characterized by a rapid loss of periodontal tissue. Several features describe AgP, such as early onset, involvement of a few or multiple teeth, and a relatively rapid progression [1,2]. There are two distinguishable patterns available: the localized form that involves the first molars and the incisors and up to two additional teeth, and the generalized form with an extensive destructive pattern [2,3,4,5]. Recently [6], the classification of the periodontal entities has been updated to stages (I–IV) and grades (A–C). The stages are based on periodontal breakdown severity, management complexity, and the extent of the disease. Grade definitions are based on the progression which in principal is related to risk factors. Practically, most of the AgP cases would be classified as stage III grade B or C.

There is a consensus that the main contributing factors are related to an impaired immune response [7], host-environment interactions and intra-host gene [1,8], and there is ethnic attribution whereas AgP is more frequent in certain geographic regions [9].

The therapy goal of AgP is to completely prevent and stop the progression of the disease, to maintain health and to regenerate the lost deprived periodontium; these goals are similar to those of chronic adult periodontitis [2]. Systematic reviews [10,11] claimed that the mechanical therapy may be as effective as the other one in both conditions.

Two different approaches may accomplish regenerative periodontal therapy: a. guided tissue regeneration (GTR) by selective cell population using tissue barriers [12] or b. enamel matrix derivates (EMD) application of tissue morphogenic factors to promote tissue growth [13,14].

Some clinical trials were performed in order to test the combined EMD/demineralized bone xenograft particles (DBX) in the belief that it may have certain qualities of a bioactive bone substitute [15,16,17,18,19,20,21,22,23,24,25,26,27]. On the other hand, previous studies that used critical size defect in rats failed to support these claims [28,29,30]. Also, in meta-analyses [31,32,33], it was not proven that there is a significant contribution of this combination. However, in 2012 Miron et al. have shown that EMD enhance osteoblast and PDL cell proliferation, differentiation and attachment to DBX particles in vitro. In vivo recent data [34] has shown that the combination between EMD and DBX particles has the ability to enhance and accelerate new bone formation in rat osseous defects.

Many randomized trials have shown encouraging results of periodontal therapy in Chronic periodontitis (ChP) patients, however, there are only a few reports that claim clinical success using either the GTR technique in AgP patients [35,36,37] or the EMD application [38,39,40,41]. Moreover, most of these reports, especially those related to the later, are based on small group of patients with no clinical standardization and/or follow-up protocols.

The aim of this this study is to retrospectively evaluate the efficacy via one of the surgical regenerative options, GTR or EMD w/wo biomaterial filler, among AgP patients in 1–10 years follow-up.

## 2. Periodontal Regeneration Procedures

### 2.1. Guided Tissue Regeneration (GTR) Procedure 

Prior to surgery, patients were admitted to rinse their mouth with 0.2% chlorohexidine, followed by local anesthesia, buccal and lingual infiltration. Muco-periosteal flaps were reflected in order to expose widely the intra-bony defects, while using the papillary preservation technique (PPT) described by Takei et al. [42,43] and Cortellini et al. [44]. in order to preserve the interproximal soft tissue. Horizontal interproximal incision was performed on the opposite side (buccal or lingual) considering the site with the deepest probing pocket depth (PPD) value. Root planning and soft tissue debridement was conducted to smooth the exposed root surface.

**Figure 1:**
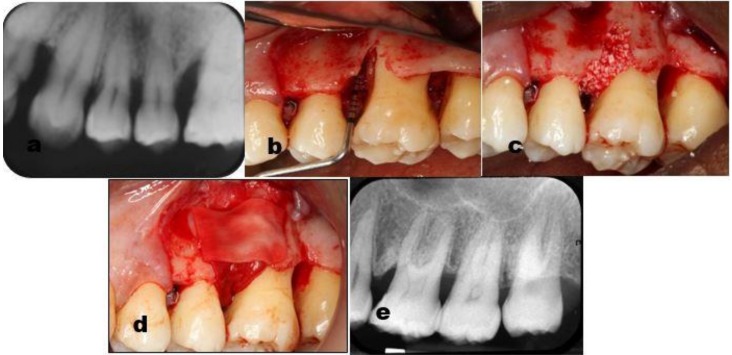
Case # 4 of the guided tissue regeneration (GTR) group, upper left sextant. The pre surgery periapical radiograph (**a**) demonstrates an extensive periodontal destruction around on the mesial aspect of the first molar. (**b**) The periodontal probe shows a 2-wall intrabony component of 7mm, which was filled by bovine bone mineral particles (**c**) and covered by a collagen membrane (**d**). two years follow-up periapical radiograph (e) shows bone filling around on the first molar.

**Figure 2:**
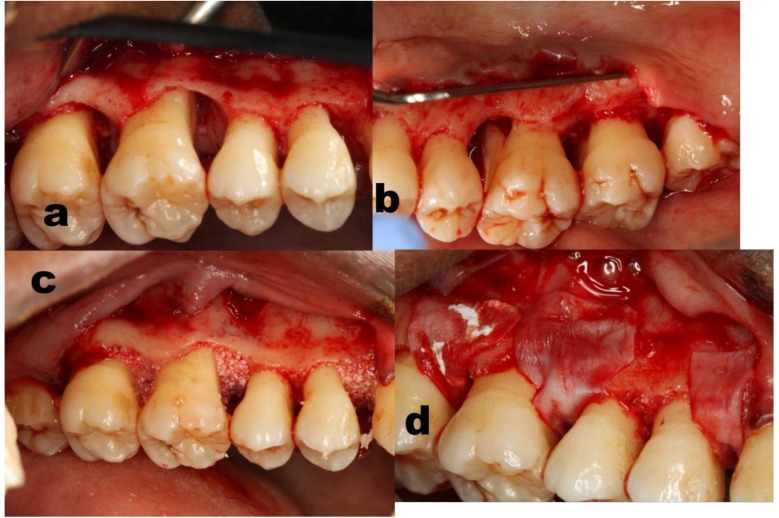
Case # 4 of the GTR group, upper right sextant. (**a**,**b**) Buccal and palatal view of the crestal bone topography. BBM particles inserted to fill the defects (**c**) followed by overlay resorbable collagen membranes (**d**).

A sample simulated matrix was trimmed to prepare a customized fit resorbable collagen membrane (Figure 1 and Figure 2, case #4). DBX particles (500–1000 µ) were placed to fill the intra bony defect, followed by coverage with the trimmed membrane. Primary soft tissue closure was achieved by releasing the flaps and stabilizing it, executing interrupted internal mattress sutures to achieve complete closure in the interproximal areas [45]. (Figure 1 and Figure 2)

### 2.2. Periodontal Regeneration by Application of Enamel Matrix Derivatives (EMD) 

Prior to surgery, patients were admitted to rinse their mouth with 0.2% chlorohexidine, followed by local anesthesia, buccal and lingual infiltration. Muco-periosteal flaps were reflected in order to expose widely the intra-bony defects, while using the PPT described by Takei et al. [42,43] and Cortellini et al. [44] in order to preserve the interproximal soft tissue. Horizontal interproximal incision was performed on the opposite side (buccal or lingual) considering the site with the deepest PPD value. Root planning and soft tissue debridement was conducted to smooth the exposed root surface.

**Figure 3:**
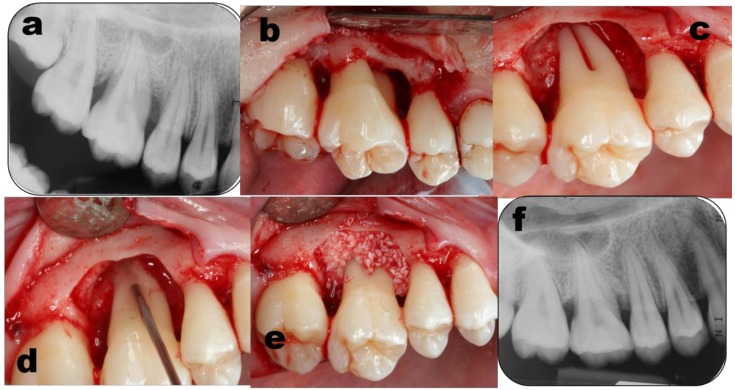
Case # 2 of the enamel matrix derivatives (EMD) group, upper right sextant. Pre surgery periapical radiograph (**a**) shows an extensive periodontal destruction around on the distal aspect of the first molar. Buccal (**b**) and palatal (**c**) aspects of the debrided roots. EMD gel was applied along the exposed roots (**d**) followed by BBM particles as a bio-material filler (**e**). four years follow-up periapical radiograph (**f**) shows bone filling around the first molar.

**Figure 4:**
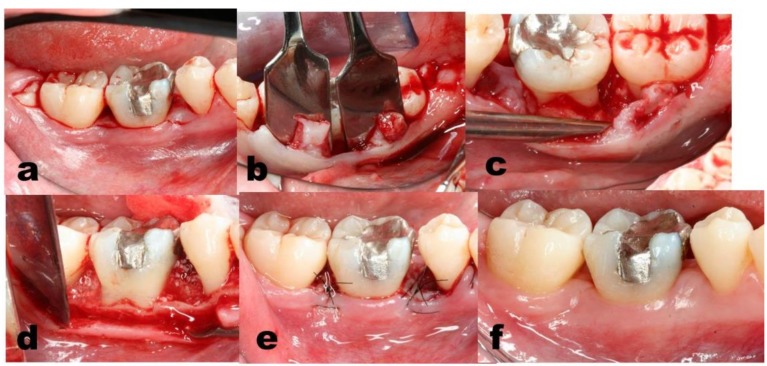
Case # 2 of the EMD group, lower right sextant. Papillary preserve technique flap elevation technique (**a**,**b**) performed to exposed the periodontal defect (**c**). EMD gel was applied on the debrided roots followed by BBM particles (**d**). In order to obtain full soft tissue closure the flaps were sutured (**e**). At 1 month, immaculate healing was evident (**f**). In order to achieve full closure, note the preservation performed of the interproximal col tissue (**b**), subsequently.

The exposed roots were conditioned with 24% EDTA for 2 minutes, followed by saline rinsing, and by applying EMD gel (Emdogain^®^) (Figure 3 and Figure 4, Case #2). Avoiding bleeding in these sites was executed. In some cases, DBX soaked in EMD gel were then added to fill the defect. Full soft tissue closure was obtained by releasing the flaps and stabilizing it, executing interrupted internal mattress sutures to achieve complete closure in the interproximal areas [45].

**Figure 5:**
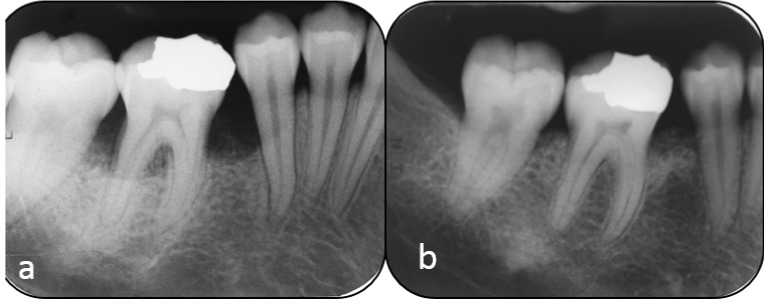
The pre (**a**) and post (**b**) periapical radiographs of Case #2 of the EMD group, lower right first molar. Note the bone filling on the mesial and distal aspect of the lower right first molar.

Strict post-op instructions were given to candidates from both groups. At 2 weeks, sutures were removed. Patients were instructed to gently clean the site with gauze soaked in CHX solution. During the maintenance phase, for the first month, patients were monitored weekly, followed by monthly visits for half a year, and once every three months later. Recall visits focused on reinforcement of oral hygiene performance and supra-gingival prophylactic cleaning. PPD, clinical attachment level (CAL) and recession height (Rec) were recorded at 6, 12, month post-surgery. Peri-apical and bite-wings radiographs were taken at the initial examination and after 6 and 12 months (Figure 3, Figure 4 and Figure 5).

### 2.3. Materials and Methods

Tel Aviv University ethics committee approved this study; 28 young (15–39 years old) healthy patients 12 males and 16 females that were diagnosed with AgP randomly selected; 6 patients (12 surgical sites) were treated by the GTR method, and therefore called the GTR group; 12 patients (54 surgical sites) were treated by the EMD method, and therefore called the EMD group.

The first appointment included extra and intra oral examinations including, a thorough periodontal chart, full mouth peri-apical radiographs and study models.

PPD, CAL, and the height of exposed roots (Rec) were recorded in all destroyed sites. As performed in previous study (Artzi et al.) [46], at each periodontally involved interproximal/inter-radicular intra-bony site, the deepest probing depth was recorded. Mean PPD and CAL (Table 1 and Table 2) represent the execution of the average measurements of all sites of each treated intra-bony defects (IBD) in each patient. For example, in a given interproximal IBD, mean probing depth was calculated as the average of the disto-buccal, disto-lingual/palatal of the mesial root (tooth) surface and the mesio-buccal and mesio-lingual/palatal of the distal root (tooth) surface. Therefore, each mean PPD site represents only the involved IBD without the neighboring unaffected shallow ones. Horizontal furcation involvement was assessed, in the inter-radicular areas [47]. The plaque score index (PI) [48] and bleeding on probing (BOP) [49] were monitored carefully. During each re-evaluation visit these parameters were repeated.

In the pre-surgical phase, the patients went through a meticulous non-surgical therapy including OHI and motivation, full mouth scaling and root debridement in conjunction followed by adjunctive systemic antibiotics of Amoxicillin 500 mg + metronidazole 250 mg (TID) for a week [50,51,52].

## 3. Results

Considering the strict oral hygiene maintenance program, patient compliance was very satisfactory. No adverse effects were noted throughout either mode of treatment. In 3 patients, a distinctive familial inheritance along their family tree was noted. However, they responded to treatment immaculately.

Periodontal indices were re-measured, upon re-evaluation of the non-surgical phase. Practically, a clinical improvement was evident as related to the periodontal indices. Table 1 and Table 2 show PPD reduction and CAL gain at follow-up, up to 10 years post the completion of the surgical phase. Since there was no significant approval on the PPD and CAL indices at the extensive IBD sites, the baseline clinical and follow-up, 10-year recordings of CAL indices are listed in the Tables.

Follow up (up to 10 years) PPD in the GTR group (n = 6; sites = 12) was reduced from 6.23 mm (±1.24 standard deviation (SD)) to 3.875 mm (±1.02). Mean PPD reduction was 2.35 mm (*p* < 0.001). Mean CAL in the GTR group reduced from 6.375 (±1.37) to 4.1 mm (±1.06); mean CAL gain was 2.27 mm (*p* < 0.001).

In the EMD group (n = 22; sites = 54), mean PPD reduced from 5.58 mm (±1.34) to 3.64 mm (±1.36). Mean reduction was 1.95 mm (*p* < 0.001). Mean CAL was reduced from 6.16 mm (±1.52) to 4.26) mm (±1.3); mean CAL gain of 1.92 mm (*p* < 0.001). Within each group, PPD reduction and CAL gain between the measurements were statistically significant.

However, tests of between subjects (GTR and EMD) effects, showed no statistical difference in regard to PPD (*p* > 0.005) nor to CAL (*p* > 0.005).

## 4. Discussion

There are two different methods to support periodontal regeneration: guided tissue regeneration (GTR) principles, and amelogenin-derived protein i.e., EMD root-surface soaking. In spite of the distinctive biological activity differences between the two methods, similar outcomes were achieved by both modalities in 1–10 years follow-up.

In the current study, GTR treated sites presented PPD reduction of 37.7% and CAL gain of 35.6%. EMD sites showed similar results with PPD reduction of 34.9% and CAL gain of 31.2%. Follow-up radiographs supported the clinical measurements, showing consistent bone augmentation and re-formation of periodontal ligament space and lamina dura ( Figure 1, Figure 3 and Figure 5).

Regeneration capacity is effected well by the IBD morphology. Thus, other shortcomings of the study design could be the fact that different IBD morphology were not considered as a significant variable factor in the interpretation of the outcome where they should be.

In order to achieve successful healing several indications are required; wound stability, re-vascularization, and the establishment of complete soft tissue closure; in regenerative treatment these are mandatory prerequisites for successful results. Furtherly, flap management via PPT should enhance the outcome of regenerative procedures [45,53]. For these reasons we used those clinical measures in both groups.

It seems that in addition to meticulous surgical execution, strict maintenance and patient compliance are key factors, regardless the surgical mode of operation.

As specified, AgP is a rapidly progressing inflammatory disease. However, devoted care may result in quite predictable long term success [11,37].

ChP and AgP have shown distinctive different etiological/contributing factors, where the later one shows an accelerated mode of aggressiveness and rapid destructiom [1]. As a consequence, the effectiveness of regenerative periodontal treatment of intrabony defects in AgP, would be of utmost importance to maintain a successful long term periodontal heaelth

It has been claimed that in severe chronic periodontitis GTR and EMD result in periodontal restitution [12,54,55,56,57,58,59,60,61,62,63,64,65,66,67,68,69,70,71,72,73]. Surprisingly, there is not enough data available regarding AgP [74,75]. However, some studies claim for successful results using either GTR procedures [35] or EMD application [36]. Enamel matrix proteins in cases of ChP seems to support wound healing and new periodontal tissue formation in IBD sites in AgP cases as well.

One can assume that although consensus reports [76] as well as systematic reviews [71] did not differentiate between IBD treatment modality of patients diagnosed with AgP and/or ChP cases, that these sites successfully healed and maintained.

If we implicate laboratory research to our research, our findings are consistent. No statistically significant differences in immunologic and microbial parameters between subjects with AgP and ChP have been presented in several reports [77,78]. Another review performed by Deas and Mealy [10] agreed that long term outcome could be comparable with blurred boundaries in ChP and Agp.

There is no accepted statement regarding the efficacy of regenerative procedures using GTR techniques in AgP patients, although few reports examined it [35,36,75,79,80], or EMD application [39,40,41,74,81,82]. In the current study we pressent a long time efficacy.

Combining GTR with a biomaterial grafting material [15,16,17,18,19,20,21,22,23,26,83] have been extensively investigated showing that the addition of a xenograft such as DBX resulted in encouraging results in IBD in ChP. Thus, this would be related to the excellent biomaterial biocompatible and conductive properties rather than to the unproven induction as shown earlier [28,29,30].

In a Cochrane systematic review, Esposito et al. [71], stated that there was no clinically significant differences between GTR and EMD in periodontal intra-bony lesions. However, it was found that the use of bone substitute materials procedures were less associated with soft tissue marginal recession compared with the application of EMD solely.

Practically, there is great significance of adding the biomaterial scaffold (DBX) whether in the GTR and/or EMD technique in order to provide maintenance of the volume of the filled defect [84]) and thus, enhance the clinical outcome.

In the EMD group, no selective barrier was used, and it can be assumed that the added biomaterial particles gave mechanical support to the soft tissue over-lay during the healing phase.

Supporting our previous study (Artzi et al.) [46] both therapy modalities are proven to achieve comparable clinical outcome i.e., stability of the soft tissue position, minimal recession and ease the ability of plaque control performance.

In conclusion, among AgP patients successful regenerative approach treatment can be achieved in a predictable manner. Whereas, the key seems to be the meticulous treatment mode for both techniques followed by strict supportive periodontal maintenance.

## 5. Conclusions

The two approaches of periodontal regeneration, guided tissue regeneration (GTR) w/wo DBX and the application of enamel matrix derivatives (EMD) w/wo DBX, achieve comparable clinical outcomes.

Successful regenerative approach treatment can be achieved predictably in aggressive periodontitis patients. The key seems to be the meticulous surgical treatment approach and a careful soft tissue flap management, for both techniques followed by a strict supportive periodontal maintenance.

## Figures and Tables

**Table 1:** GTR group, pre-op and follow-up periodontal probing pocket depth (PPD) mean measurements.

Site	PPD pre	CAL pre	PPD curr	CAL curr	PDR ^1^	CAL GAIN
1	5.5	5.5	5	5	0.5	0.5
2	6.5	7	3.5	4.25	3	2.75
3	4	4	2	2	2	2
4	5.5	5.5	5.5	5.5	0	0
5	4.5	4.5	5.25	5.5	−0.75	−1
6	7	7.5	4.75	4.75	2.25	2.75
7	6.5	6.5	3.5	3.5	3	3
8	6	6	3.75	3.75	2.25	2.25
9	7.5	8	2.75	2.75	4.75	5.25
10	8.5	8.75	3	3.25	5.5	5.5
11	7.5	7.5	3.5	5	4	2.5
12	5.75	5.75	4	4	1.75	1.75
Average ^2^	6.23	6.37	3.87	4.10	2.36	2.27
SD	1.24	1.37	1.02	1.06	1.79	1.82

^1^ PDR—probing depth reduction. ^2^ The average of 4 probing depth measurements at the disto-buccal, mesio-buccal, mesio-lingual and disto-lingual of each given intra-bony defects (IBD) site.

**Table 2:** EMD group, pre-op and follow-up periodontal probing pocket depth (PPD) mean measurements.

site	PPD pre	CAL pre	PPD curr	CAL curr	PDR ^1^	CAL GAIN
1	5	5.5	3	3.5	2	2
2	4.75	5.75	2.25	3.75	2.5	2
3	3.75	3.75	3	3.75	0.75	0
4	7	7	2.75	3.75	4.25	3.25
5	4	4	4.5	4.5	−0.5	−0.5
6	6.5	6.5	4.25	4.25	2.25	2.25
7	4.5	4.5	3.75	3.75	0.75	0.75
8	5.5	5.5	6.5	6.5	−1	−1
9	5.5	5.5	3.75	3.75	1.75	1.75
10	5	5	3	3	2	2
11	6	6.75	6	6.75	0	0
12	8	8	6.5	7.25	1.5	0.75
13	7	7	3.25	4.5	3.75	2.5
14	6.5	9	2	4	4.5	5
15	5	6.5	4	6	1	0.5
16	7.5	10.25	3.25	5.75	4.25	4.5
17	4.5	6	3.25	4.25	1.25	1.75
18	5.75	5.75	2.25	2.25	3.5	3.5
19	5	5	3.5	3.5	1.5	1.5
20	7.5	8	6	6	1.5	2
21	4.5	5	4.5	4.5	0	0.5
22	7.5	7.5	3	4	4.5	3.5
23	3.5	5.25	2	3.25	1.5	2
24	5.5	5.5	7	7	1.5	−1.5
25	6.5	7	6.25	6.75	0.25	0.25
26	4	4.5	2.5	4	1.5	0.5
27	4.5	5	3.5	3.5	1	1.5
27	5.25	7.25	2.5	3.5	2.75	3.75
29	4	6.5	3	5.25	1	1.25
30	4.75	6	2	2.5	2.75	3.5
31	7	8	3.25	3.75	3.75	4.25
32	4.75	4.75	3.25	4.25	1.5	0.5
33	6	6	3.5	4.5	2.5	1.5
34	6.75	7.5	4.75	5.75	2	1.75
35	6.25	7.25	5.25	5.75	1	1.5
36	6.75	6.75	4	4	2.75	2.75
37	6.75	6.75	4	4	2.75	2.75
38	6.5	6.75	7	7	–0.5	–0.25
39	5.5	6	extracted			
40	8.75	9	3	3	5.75	6
41	8.5	8.75	3	3	5.5	5.75
42	3.75	3.75	2	3	1.75	0.75
43	6	6	2	3	4	3
44	4.75	5	2.25	2.75	2.5	2.25
45	5.5	6.5	2	2.5	3.5	4
46	7	9	3.25	4.25	3.75	4.75
47	4	5.75	5.25	5.75	–1.25	0
48	4.25	4.25	3	3.5	1.25	0.75
49	4	5	2.75	3.75	1.25	1.25
50	4.25	4.25	3.5	3.5	0.75	0.75
51	4	4	3.25	3.25	0.75	0.75
52	3.5	3.5	2.75	2.75	0.75	0.75
53	5.75	5.75	3	3.25	2.75	2.5
54	7	7.75	3.75	3.75	3.25	4
Average ^2^	5.58	6.16	3.64	4.24	1.94	1.92
SD	1.34	1.52	1.35	1.3	1.64	1.68

^1^ PDR—probing depth reduction. ^2^ The average of 4 probing depth measurements at the disto-buccal, mesio-buccal, mesio-lingual and disto-lingual of each given IBD site.
